# Evaluation of Steaming and Drying of Black Sesame Seeds for Nine Cycles Using Grey-Correlation Analysis Based on Variation-Coefficient Weight

**DOI:** 10.3390/molecules28135266

**Published:** 2023-07-07

**Authors:** Yongqing Zhang, Jiaojiao Wang, Huihui Tan, Xinyue Lu, Deguo Wang, Quanzeng Wei

**Affiliations:** Key Laboratory of Biomarker—Based Rapid Detection Technology for Food Safety of Henan Province, Xuchang University, Xuchang 461000, China; zyq336@163.com (Y.Z.);

**Keywords:** black sesame, nine steaming nine drying, high-pressure steaming, grey-correlation degree, variation-coefficient method

## Abstract

This study aimed to improve the steaming process of black sesame seeds. A comprehensive evaluation was conducted using the grey-correlation method based on the variation-coefficient weight to observe the treatments of normal-pressure (NPS) and high-pressure (HPS) steaming (with/without soaking in water) for nine cycles. Their effects on the contents of water, protein, fat, ash, melanin, sesamin, and sesamolin of black sesame seeds, as well as the sensory score of the black sesame pill, were determined. We found that with varied steaming methods and increased steaming cycles, the contents of the nutritional and functional components of black sesame seeds and the sensory score of the black sesame pill differed. The results of the variation-coefficient method showed that water, protein, fat, ash, melanin, sesamin, sesamolin, and sensory score had different effects on the quality of black sesame seeds with weighting factors of 34.4%, 5.3%, 12.5%, 11.3%, 13.9%, 11.3%, 7.8%, and 3.5%, respectively. The results of two-factor analysis of variance without repeated observations indicated that the grey-correlation degree of HPS was the largest among the different steaming treatments, and the following sequence was HPS after soaking in water (SNPS), NPS, and SNPS. There was no significant difference between NPS and SNPS (*p* < 0.05). Moreover, with increased cycles, the value of the grey-correlation degree increased. The comprehensive score of the procedure repeated nine times was significantly higher than other cycles (*p* < 0.05). The results of the grey-correlation degree and grade analysis showed that the best steaming process of black sesame seeds was HPS for nine cycles, followed by HPS for eight cycles and NPS after soaking in water (SNPS) for nine cycles. These findings could provide a scientific basis for replacing SNPS with HPS to simplify steaming and realize the parametric steaming of black sesame seeds, and thus, ensure the quality of black-sesame products.

## 1. Introduction

Sesame (*Sesamum indicum* L.) of the Pedaliaceae family is an important and ancient oilseed crop with more than 50% oil [1] and 20% proteins [2]. Sesame seeds are also rich in carbohydrate, minerals, fat-soluble vitamins, and some secondary metabolites, such as flavonoids, saponins, and phenolic compounds [3]. Sesame oil also contains more than 80% linoleic and linolenic acids and is more resistant to oxidative changes than other vegetable oils due to lignans, such as sesamin, sesamolin, and sesamol [4]. These bioactive compounds confer sesame and sesame products with special health benefits such as protection against inflammation, cancer, hypocholesterol, coronary artery disease, and others chronic diseases [1]. Due to the high nutritional value, unique flavor, and good medical practice of sesame and sesame products, they are eaten as a kind of healthy food in Eastern and Western worlds [5,6,7]. However, some antinutrients (e.g., phytate and oxalate) also exist in sesame. The antinutrients and hard shells of sesame seeds cause raw sesame seeds to be poorly absorbed and digested in humans and also reduce the bioavailability of minerals [8]. Consequently, sesame seeds are commonly processed before consumption through various processing methods, such as roasting, steaming, drying, and stir frying [9].

Nine steaming–nine drying (NSND) is an ancient and traditional processing method that involves nine cycles of steaming and drying. It is commonly utilized to process food and medicinal ingredients in China [10]. NSND reportedly changes the overall carbohydrates, polysaccharide, and secondary metabolites of Rehmanniae radix significantly [11]. NSND further turns ginsenosides into low-polarity constituents that benefit the antiviral potency of ginsenosides [12,13]. Nevertheless, the ancient and traditional processing technology of NSND is so complex and long that it is not used anymore in some processing enterprises. To easily operate, some manufacturers even give up the special NSNS and instead choose other simple processes (e.g., stir frying). However, different processing methods have different effects [14]. The results of a comparison and analysis of antioxidant activity of black soybean soaked in vinegar have shown that the order of antioxidant activity decreases as roasting, stir-frying, raw black beans without treatment, high pressure, and boiling [15]. Accordingly, the quality of product prepared with the different processing methods differed on the market. To ensure processing effect and product quality, improving the processing technology of NSND is urgent.

High-pressure steaming (HPS) is a process for rapidly ripening grain by using saturated steam at high pressure [16]. The processing effects of some traditional Chinese medicinal herbs (e.g., *Cistanches Herba* and Radix Aconiti) have proven that HPS technology could meet the requirement of corresponding products, save time, and be simple to operate [17]. HPS is also a good hydrothermal treatment for lipid recovery and does not significantly change the fatty acid profile in lipid extraction from *Chlorella vulgaris* [14]. However, the application of HPS in processing black sesame seeds has not been reported.

Soaking seeds is a process in which seeds absorb water and swell simultaneously, thereby softening the seed coat to facilitate processing. After soaking, grain hardness decreases and other physical properties of minor millets (e.g., bulk density, true density, porosity, and coefficient of static friction) change [18]. Soaking can also remove some antinutritional factors of food materials to improve the nutritional value [19]. Before NSND, soaking is utilized in the actual production process of black sesame seeds by a factory named Yuzhou Hou Sheng Tang Chinese Medicine Co., Ltd. (Yuzhou, China). Actually, no work on the necessity of soaking treatment has been reported, and the processors primarily rely on trial to achieve the desired level of steaming black sesame seeds.

As a traditional Chinese health food, nine steamed–nine sun-dried black sesame seeds are regarded as a top-grade tonic [20]. A processing method is the basis for black sesame seeds to realize the modernization of technology and the standardization of quality. To provide a scientific basis for wisely selecting the effective processing method, determining the potential factors that affect the quality of black sesame seeds is necessary. The large amount of data obtained from various processing technologies is cumbersome and complex. The grey-correlation method can be used to determine the uncertain relations among factors or their contribution to a given system by calculating the absolute value of the data difference between sequences [21]. This method has been utilized to optimize multi-objectively the Q355C steel gas metal arc-welding process [22] and the process parameters for plastic injection molding [23]. The correlation degrees between the frost-penetration depths and different parameters, including thermal characteristics, average annual temperatures, and so on have also been presented by grey-correlation analysis [24]. The present study focused on determining the main nutritional and functional components and product sensory score of black sesame seeds processed for nine cycles by normal-pressure steaming (NPS) or HPS (with/without soaking treatment at water for 24 h). The experimental data were analyzed and compared. Then, the importance of different steaming technologies affecting the quality indicators of black sesame seeds was sorted by the grey-correlation method based on the variation-coefficient weight. Our findings may provide information and a comprehensive-evaluation approach to improving the steaming process of black sesame seeds and ensuring product quality.

## 2. Results

### 2.1. Effect of Various Steaming Processes on the Quality Indicators of Black Sesame Seeds

Black sesame seeds were treated for nine cycles with the process of SNPS, NPS, SHPS or HPS, respectively. Table 1, Table 2, Table 3 and Table 4 show that the quality indicators of black sesame seeds had a relatively high sensitivity to the changes in processing methods and processing cycles. With increased processing cycles, the contents of water, melanin, sesamin, and sesamolin decreased basically. When the procedure was repeated for nine cycles, the comparison between steaming methods affecting the quality of black sesame seeds is shown in Table 5 (*p* < 0.05). Except for ash content, other indicators of black sesame seeds showed significant differences among the four processes.

### 2.2. Weight Value of Each Influencing Factor

Using Equations (1) and (2), *Vi* and *Wi* were calculated and are shown in Table 6. Based on the different contributions of these indicators, the weights of water, protein, fat, ash, melanin, sesamin, sesamolin, and sensory score were 34.4%, 5.3%, 12.5%, 11.3%, 13.9%, 11.3%, 7.8%, and 3.5%, respectively. The highest weight of 34.4% indicated that water content exerted the greatest change in black sesame seeds throughout the four processes.

### 2.3. Nomalization of Raw Data

To meet quality targets, selecting the processing technologies with high contents of nutritional and functional components and excellent sensory properties for black-sesame products was desirable. Therefore, in the ideal process selected as the reference sequence (X_0_), the protein, fat, ash, melanin, sesamin, sesamolin, and sensory score were the highest in the corresponding indicators of SNPS, NPS, SHPS, and HPS. In terms of moisture, with increased processing cycles, the contents of water decreased (Table 1, Table 2, Table 3 and Table 4). And NSND that involves nine cycles of steaming and drying indicated that the lower the water content, the better the treatment effect. Thus, in the ideal process selected as the reference sequence (X_0_), the water was the lowest in the water contents of SNPS, NPS, SHPS, and HPS. All raw data were normalized with Equation (3). The standardized data of evaluation indices are listed in Table 7.

### 2.4. Absolute Difference between Comparison and Reference Sequence

The absolute differences between comparison and reference sequence were calculated with Equation (4) and are shown in Table 8.

According to Equations (5) and (6), the minimum absolute difference was 0.000, and the maximum absolute difference was 7.040.

### 2.5. Grey-Correlation Coefficient

Equation (7) was used to count the correlation coefficient between the steaming technologies (SNPS, NPS, SHPS, and HPS) and the quality indicators of black sesame seeds. Then, the calculated correlation coefficients are given in Table 9.

### 2.6. Grey-Correlation Degree and Grade

**Figure 1:**
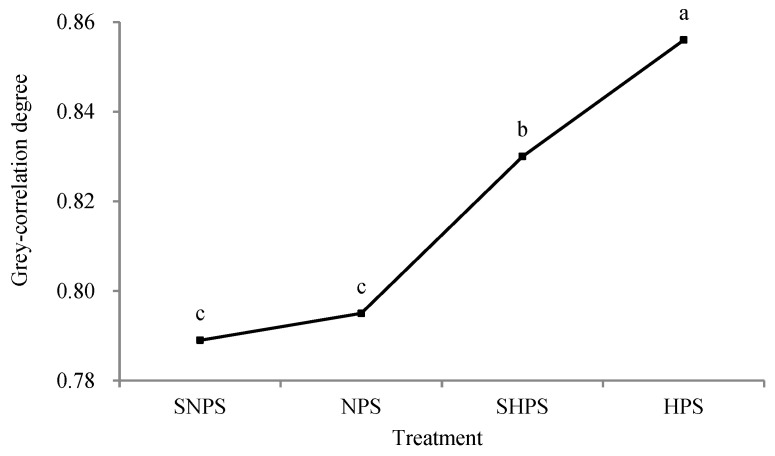
Results of analysis of variance between treatments and grey-correlation degree. Spots marked by a different letter are significantly different (*p* < 0.05).

**Figure 2:**
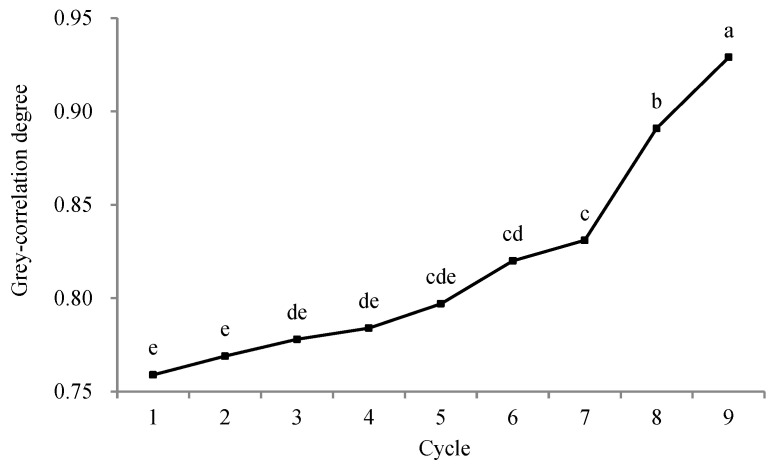
Results of analysis of variance between cycles and grey-correlation degree. Spots marked by a different letter are significantly different (*p* < 0.05).

The grey-correlation degrees calculated with Equation (8) and the correlation grades (including the correlation grade within a group and correlation grade between groups) are given in Table 9. Two-factor analysis of variance without repeated observations was used to investigate the relation between grey-correlation degree and the processing technology (Figure 1 and Figure 2).

According to the correlation grade within a group, with increased cycles, the value of grey-correlation degree during HPS, SHPS, SNPS, and NPS generally increased. The procedure repeated nine times was the optimum, with the statistically highest comprehensive score of black sesame seeds among the four methods (Figure 2). This finding also indicated that the traditional processing method of NSND involving nine cycles of steaming and drying can reasonably process food and medicinal ingredients in China.

Based on the correlation grade between groups, the top three samples in terms of grey-correlation degree in decreasing order were X_36_, X_35_, and X_9_. Thus, the combined score of the sample during SNPS repeated steaming for nine cycles was lower than those during HPS repeated steaming for eight and nine cycles. Comparing the four processes (Table 9 and Figure 1), the comprehensive score of HPS with/without soaking in water was significantly higher than that of NPS or SNPS. Therefore, HPS could be used to replace SNPS and simplify the steaming process to realize the parametric steaming of black sesame seeds.

## 3. Discussion

Melanin is a photoprotective pigment which functions as an antioxidant, free-radical scavenger, and charge-transport mediator [25]. Sesamin and sesamolin are two lignans present in sesame seeds with some functions, such as antioxidant, decreasing blood lipids, and anti-inflammatory [26]. Considering the advantages of melanin, sesamin, and sesamolin on human health, selecting the processing technologies with high contents of melanin, sesamin, and sesamolin for black-sesame products was desirable. Our results indicated that fewer treatments were better for melanin, sesamin, and sesamolin during the steaming and drying of black sesame seeds (Table 1, Table 2, Table 3 and Table 4).

Table 5 shows that the processes with/without soaking in water led to different water contents of black sesame seeds (e.g., 5.09% of SNPS vs. 11.37% of NPS, 8.64% of SHPS vs. 4.92% of HPS). In other words, different effects were observed on the water contents of black sesame seeds by soaking before NPS and HPS. Soaking black sesame seeds could effectively reduce water content during NPS, but the opposite result was obtained during HPS. The reason for the promoted water reduction of black sesame seeds soaking during NPS may be that soaking makes seeds absorb water and swell simultaneously, and can soften the seed coat to facilitate transferring heat to reduce the water of black sesame seeds. Nevertheless, HPS can achieve steaming at high pressure to break the seed coat by providing sufficient pressure to vaporize water inside the seed, and the hard seed coat should be more sensitive to high pressure than the soft seed coat. Therefore, the coat-breaking effect of HPS on unsoftened black sesame seeds was better than that on softened ones such that water in the seeds was more effectively evaporated. This finding was confirmed by the significantly lower water content of HPS than SHPS (Table 5).

Meanwhile, water absorption in seeds’ soaking process increased the seed moisture content so that more water needed to be removed in the steaming and drying of black sesame seeds. If the processing time was insufficient, the increase in moisture content cannot be decreased. In the first 6 cycles, the water content of SNPS or SHPS was higher than that of NPS or HPS (Table 1, Table 2, Table 3 and Table 4). Steaming times must exceed six cycles for SNPS or SHPS to remove absorbed water in black sesame seeds. To reduce the adverse effects of the antinutrients and hard shells of sesame seeds and thus improve the bioavailability of minerals, processes such as roasting, steaming, drying, and stir-flying are commonly utilized before sesame-seed consumption [8,9]. Certainly, the moisture content of sesame seeds during these processes showed a downward trend. After processing, the water was less than 7.5%, which is commonly required for the long-term storage of sesame. Considering water content, the nine cycles of black sesame seeds treated with SNPS in actual production by Yuzhou Hou Sheng Tang Chinese Medicine Co., Ltd. (Yuzhou, China) were reasonable, and SNPS could be replaced by HPS because the 4.92% water content of HPS was statistically equal to the 5.09% of NSND with soaking in water (SNPS).

Based on the determinations of the quality indicators, such as water, protein, fat, ash, melanin, sesamin, sesamolin, and sensory score, the results of comprehensive evaluation using grey-correlation degree and grade analysis showed that the best steaming process of black sesame seeds was HPS for nine cycles. The effect of HPS on the black sesame seeds in our study was identical with the role of the high-pressure cooking method in the extraction of corn germ protein. The extraction effect of protein digestion in corn germ meal treated with HPS, and the high-pressure steam treatment significantly reduces the processing time, better than the soaking treatment of raw materials [27]. These findings could provide a scientific basis for replacing SNPS with HPS to simplify steaming and realize the parametric steaming of black sesame seeds, and thus, ensure the quality of black-sesame products. Furthermore, with the development of drying technology, such as infrared radiation drying [28] and advanced osmotic dehydration techniques combined with emerging drying methods [29], the drying method used in the NSND should be studied further to promote the application of NSND.

## 4. Materials and Methods

### 4.1. Raw Materials

Black sesame seeds, honey, and sealwort juice were provided by Yuzhou Hou Sheng Tang Chinese Medicine Co., Ltd. (Yuzhou, China). Black rice, black soya bean, white glutinous rice, dry mulberry, dry wolfberry, and dry haw were purchased from a local market in Xuchang, Henan, China. The materials were cleaned by hand picking and winnowing to remove foreign matter.

### 4.2. Steaming Technologies

(1) NPS after soaking in water (SNPS). First, the cleaned black sesame seeds were soaked in tap water for 24 h and then the water was drained. Second, the seeds were steamed for 2 h over boiling water in an electric steamer (Huidang Home, 34 cm, Shandong Huidang Home Appliances Co., Ltd., Jinan, China) for which the power was set to 1800 w. Then, the seeds were dried for 2 h in an oven (101-2AB, Beijing Zhongxing Weiye Instrument Co., Ltd., Beijing, China) at 60 °C, and stirred once every 30 min. The steaming and drying procedure was repeated for nine cycles to collect nine samples of SNPS denoted as X_1_–X_9_, respectively.

(2) NPS. Without soaking, the seeds were steamed and dried for nine cycles, following the same steps as the above section of “NPS after soaking in water (SNPS)”, to collect nine samples of NPS denoted as X_10_–X_18_, respectively.

(3) HPS after soaking in water (SHPS). The SHPS processing also involved three steps with the same as the above section of “NPS after soaking in water (SNPS)”, but the second step differed. During SHPS, the black sesame seeds were steamed for 30 min at 121 °C in a high-pressure steam sterilizer (YXQ-SG46-280SA, Shanghai Boxun Industry Co., Ltd., Shanghai, China) with 0.21 Mpa pressure. The steaming and drying procedure was repeated for nine cycles to collect nine samples of SHPS denoted as X_19_–X_27_, respectively.

(4) HPS. Without soaking, the seeds were steamed and dried for nine cycles, following the same steps as the above section of “HPS after soaking in water (SHPS)”, to collect nine samples of HPS denoted as X_28_–X_36_, respectively.

### 4.3. Nutritional and Functional Analysis of Black Sesame Seeds

The moisture content was measured using the method of direct drying described by GB 5009.3-2016 (China). Crude protein was determined using the Kjeldahl method described by GB 5009.5-2016 (China). Fat was detected by the Soxhlet extraction method described by GB 5009.6-2016 (China). Total ash was determined by the first method described by GB 5009.4-2016 (China). Each sample was set to have three parallels.

Melanin was detected with an ultraviolet photometer (Bluestar, Beijing Labtek Instrument Co., Ltd., Beijing, China) at 360 nm. Alcohol was used to extract black sesame pigments, and the extraction technology conditions were as follows: 50% alcohol (*v*/*v*), 1:50 ratio of black sesame seeds to solvent, pH 1.0, and extraction for 30 min. Each sample was set to have three parallels.

Sesamin and sesamolin were measured using a high-performance liquid chromatography with UV detector (Agilent1260, Agilent Technology Co., Ltd., Palo Alto, CA, USA) described by NY/T 1595-2008 (China). The chromatographic conditions of HPLC were Agilent XDB-C18 column (4.6 mm × 250 mm, 5 μm); column temperature 30 °C; mobile phase: CH_3_OH/H_2_O = 80/20; flow rate 0.8 mL/min; and wavelength 290 nm. Each sample was tested twice. The curves of sesamin and sesamolin standards were shown in Supplementary File S1: Figures S1 and S2.

### 4.4. Sensory Evaluation of Black-Sesame Product

Black-sesame products include black-sesame paste, black-sesame pill, black-sesame cake, and so on. Among them, the black-sesame pill is a kind of honeyed pill using the steamed black sesame seeds as their main raw materials. To measure the impact of steaming and drying on the quality of the black-sesame product, the black-sesame pill was prepared with the black sesame seeds exposed to the different steaming technologies in the Section 4.2, and sensory evaluation was conducted.

(1) Blend formulation and preparation of the black-sesame pill

Raw material flours. First, black rice, black soya bean, white glutinous rice, and the steamed black sesame seeds were separately fried with incense. Second, the fried raw materials, mulberry, wolfberry, and haw were milled using a stainless universal crusher and sieved into fine flour of 40 mesh, respectively.

Refining honey. No-crystal honey was boiled for 4 s on an induction cooker at 100 °C, and the boiling procedure was repeated three times.

Blend formulation. The basic formulation of the black-sesame pill used was as follows: black sesame flour, 30 g; the refined honey, 16 g; sealwort juice, 2 g; black rice flour, 1 g; black soya bean flour, 1 g; mulberry flour, 1 g; wolfberry flour, 1 g; wolfberry flour, 1 g; white glutinous rice flour, 1 g; haw flour, 0.4 g; and sodium carboxymethyl cellulose, 0.3 g.

Black-sesame pill preparation. The dry ingredients were weighed and thoroughly mixed. Sealwort juice was added and mixed until uniformity was achieved. The refined honey was added and kneaded into a ball while it was hot, and hand beaten 60 times. Then, the ball was weighed in groups. A black-sesame pill was 8 g. The pill of 8 g was initially hand rubbed in 20 circles to a smooth surface without cracks. Then, the rubbed pill was dried for 30 min in an oven at 60 °C, and flipped once at 15 min. Finally, the pill taken out from the oven was cooled to room temperature and packed using a silver paper of 8 cm × 8 cm. This was the finished product.

(2) Sensory evaluation of black-sesame pill

Quality indicators of black-sesame pill samples were evaluated by a panel of 10 students majoring in food according to the sensory scoring criteria (Supplementary File S2: Table S1). Sensory attributes of food morphology, color, texture and flavor were graded by percentage points.

The sensory evaluation process was as follows. First, the shape and color of the prepared black-sesame pill which was placed on a white plate were observed; second, the pill was smelt; third, tasted; then, the average of each factor was calculated; finally, the sensory score was calculated using a weighted scoring method, in which the weights of morphology, color, texture and flavor were 20%, 22%, 25% and 33%, respectively.

### 4.5. Variation-Coefficient Weighting Method

The variation-coefficient method can avoid equal division of weight and make results more reasonable [30]. The steps are listed below.

(1) Calculating the variation-coefficient of indicator.

(1) $$V_{i}=\frac{\delta_{i}}{X_{i}}\;$$

where $V_{i}$ is the variation-coefficient of the indicator *C_i_*; $\delta_{i}$ is the standard deviation of the indicator *C_i_*; $X_{i}$ is the mean value of the indicator *C_i_*; *i* = 1, 2, …, *n*; and *n* is the number of the indicators, and *n* = 1, 2, …, 8.

(2) Calculating objective weight.

(2) $$W_{i}=\frac{V_{i}}{\sum_{i=1}^{n}V_{i}}$$

where *W_i_* is the object weight of the indicator *C_i_*; *V_i_* is the variation-coefficient of the indicator *C_i_*; and *n* is the number of indicators (*n* = 1, 2, …, 8).

### 4.6. Grey-Correlation Analyses

The grey-correlation analysis is a geometric approach used to evaluate the correlation between different sequences within a design [24]. To eliminate the dimensional differences of the various indicators, the influences of various steaming processes on the quality indicators of black sesame seeds were evaluated by grey-correlation analysis based on the contents of water, protein, fat, ash, melanin, sesamin, and sesamolin of black sesame seeds, and the sensory score of the black sesame pill. The following steps were included in grey-correlation analysis.

(1) Normalizing raw data. The ideal process was selected as the reference sequence (*X*_0_), and the other processing methods (SNPS, NPS, SHPS, and HPS) were taken as the comparison sequence (*X_j_*). Except for the minimum water content, the other quality indicators of the ideal process were the highest in the corresponding indicators of SNPS, NPS, SHPS, and HPS. The equalization method was used to preprocess the raw data and convert them into dimensionless data [31].

(3) $$\;C_{i}^{’}\left(k\right)=\frac{C_{i}\left(k\right)}{c_{0}\left(k\right)\;}\;$$

where *i* is the number of the samples (*i* = 0, 1, 2, …, 36); and *k* is the number of indicators (*k* = 1, 2, …, 8).

(2) Determining the absolute difference between the comparison and the reference sequence. The absolute difference was calculated using Equation (4) [32].

(4) $$\;\Delta_{i}\left(k\right)=|C_{0}^{’}\left(k\right)-C_{i}^{’}(k)|\;$$

The minimum absolute difference was as follows:

(5) $$\begin{array}{c} \Delta min=minmin|C_{0}^{’}\left(k\right)-C_{i}^{’}\left(k)\right|\\ i\;\;\;\;k\; \end{array}$$

The maximum absolute difference was as follows:

(6) $$\begin{array}{c} \Delta max=maxmax|C_{0}^{’}\left(k\right)-C_{i}^{’}\left(k)\right|\\ i\;\;\;\;k\; \end{array}$$

where *i* is the number of samples (*i* = 0, 1, 2, …, 36); and *k* is the number of indicators (*k* = 1, 2, …, 8).

(3) Calculating the grey-correlation coefficient. The grey-correlation coefficient is defined as the correlation between the ideal and actual standardized value. It can be calculated using Equation (7) [32].

(7) $$\zeta_{i}\left(k\right)=\frac{\Delta min+\rho \Delta max}{\Delta_{i}\left(k\right)+\rho \Delta max}$$

where *i* is the number of the samples (*i* = 0, 1, 2, …, 36); *k* is the number of indicators (*k* = 1, 2, …, 8); and ρ is the resolution ratio, which serves to enlarge the difference between various coefficients, and ρ = 0.5 is generally used.

(4) Calculating grey-correlation degree and arranging grey-relational grade. To obtain the grey-relational degree, the following Equation (8) was used:

(8) $$X_{i}=\overset{k}{\underset{i=1}{\sum }}\zeta_{i}\left(k\right)\times W_{i}\;{\left(k\right)}_{}$$

where $\zeta_{i}\;\left(k\right)$ is the grey-correlation coefficient of the indicator *C_i_*; $W_{i}\;\left(k\right)$ is the object weight of the indicator *C_i_*; *i* is the number of the samples, and *i* = 0, 1, 2, …, and 36; *k* is the number of the indicators, and *k* = 1, 2, …, 8.

The grey-relational grade *R_i_* is the sequence arranged by the value of grey-correlation degree of the samples.

### 4.7. Statistical Analyses

Experimental and sensory results were subjected to analysis of variance, and means were compared using the Student–Newman–Keuls test. Significance was accepted at *p* ≤ 0.05.

## 5. Conclusions

The four steaming processes of SNPS, NPS, SHPS, and HPS exerted different degrees of influence on the nutritional and functional ingredients of black sesame seeds and the sensory score of the black sesame pill. The influences of various steaming processes on the quality indicators of black sesame seeds were evaluated using grey-correlation analysis by analyzing the contents of water, protein, fat, ash, melanin, sesamin, and sesamolin of black sesame seeds and the sensory score of the black sesame pill. We found that nine cycles of black sesame seeds treated with steaming and drying was the optimum. Moreover, the traditional technology of NSND was reasonable. These findings indicated that HPS, as a new production process of black sesame seeds, was suitable for replacing SNPS, and the soaking before steaming process could be avoided. Overall, HPS could save time and simplify SNPS steps. The required equipment was also simple and desirable for the parametric steaming and large-scale production of black-sesame products. However, the effects of these processes on other components (e.g., amino acid and fat acid) in black sesame seeds were not observed. Therefore, further research is needed to assess the processing effect of black sesame seeds by examining other components, especially essential amino acids and essential fatty acids, to provide an idea for the study of traditional and modern processing technology. Overall, a method of comprehensive analysis was established to determine the effects of different processing technologies on black sesame seeds by analyzing multiple indicators. This study provided a scientific basis for the further development and utilization of new technologies.

## Figures and Tables

**Table 1:** Effect of processing cycles on the quality indicators of black sesame seeds treated with SNPS.

Sample	Cycle	Water (g/100 g)	Protein (g/100 g)	Fat (g/100 g)	Ash (g/100 g)	Melanin (Abs)	Sesamin (mg/g)	Sesamolin (mg/g)	Sensory Score (Point)
X_1_	1	39.54 ± 0.31 ^a^	21.92 ± 0.25 ^b^	33.96 ± 0.46 ^e^	2.93 ± 0.02 ^g^	0.775 ± 0.003 ^a^	4.49 ± 0.01 ^a^	2.29 ± 0.00 ^a^	78.36 ± 0.88 ^f^
X_2_	2	36.23 ± 0.61 ^b^	21.17 ± 0.44 ^c^	34.15 ± 0.66 ^e^	3.48 ± 0.06 ^f^	0.704 ± 0.002 ^b^	4.25 ± 0.00 ^d^	2.16 ± 0.00 ^d^	79.26 ± 0.83 ^e^
X_3_	3	35.36 ± 0.48 ^c^	20.31 ± 0.17 ^d^	34.91 ± 0.66 ^e^	3.74 ± 0.03 ^e^	0.695 ± 0.002 ^c^	4.45 ± 0.00 ^b^	2.25 ± 0.00 ^b^	80.64 ± 0.84 ^d^
X_4_	4	30.00 ± 0.20 ^d^	19.71 ± 0.07 ^d^	36.55 ± 0.40 ^d^	3.55 ± 0.01 ^f^	0.576 ± 0.003 ^d^	4.23 ± 0.00 ^e^	2.14 ± 0.00 ^e^	83.68 ± 0.66 ^c^
X_5_	5	29.60 ± 0.11 ^d^	19.81 ± 0.11 ^d^	42.01 ± 0.61 ^c^	3.70 ± 0.02 ^e^	0.544 ± 0.003 ^e^	4.28 ± 0.00 ^c^	2.18 ± 0.00 ^c^	84.12 ± 0.95 ^c^
X_6_	6	23.22 ± 0.14 ^e^	19.64 ± 0.16 ^d^	43.67 ± 0.19 ^b^	3.94 ± 0.05 ^d^	0.535 ± 0.003 ^f^	3.71 ± 0.00 ^g^	1.91 ± 0.00 ^g^	85.33 ± 0.73 ^b^
X_7_	7	22.56 ± 0.71 ^e^	24.33 ± 0.54 ^a^	44.77 ± 0.47 ^a^	4.52 ± 0.10 ^c^	0.516 ± 0.004 ^g^	3.49 ± 0.00 ^i^	1.85 ± 0.00 ^h^	87.06 ± 0.61 ^a^
X_8_	8	11.19 ± 0.29 ^f^	22.27 ± 0.47 ^b^	43.48 ± 0.11 ^b^	5.09 ± 0.13 ^b^	0.505 ± 0.001 ^h^	3.72 ± 0.00 ^f^	1.95 ± 0.00 ^f^	86.95 ± 0.44 ^a^
X_9_	9	5.09 ± 0.44 ^g^	21.25 ± 0.20 ^c^	42.10 ± 0.26 ^c^	5.24 ± 0.05 ^a^	0.505 ± 0.002 ^h^	3.58 ± 0.00 ^h^	1.91 ± 0.00 ^g^	85.14 ± 0.90 ^b^

Values (mean ± SD) in each column followed by a different letter are significantly different (*p* < 0.05); SNPS: normal-pressure steaming after soaking in water.

**Table 2:** Effect of processing cycles on the quality indicators of black sesame seeds treated with NPS.

Sample	Cycle	Water (g/100 g)	Protein (g/100 g)	Fat (g/100 g)	Ash (g/100 g)	Melanin (Abs)	Sesamin (mg/g)	Sesamolin (mg/g)	Sensory Score (Point)
X_10_	1	32.79 ± 0.31 ^a^	22.87 ± 0.17 ^a^	33.81 ± 0.41 ^h^	3.49 ± 0.01 ^g^	0.786 ± 0.003 ^a^	5.03 ± 0.00 ^a^	2.53 ± 0.00 ^a^	80.33 ± 0.66 ^h^
X_11_	2	26.23 ± 0.04 ^b^	21.05 ± 0.43 ^bc^	33.42 ± 0.26 ^h^	3.84 ± 0.05 ^e^	0.739 ± 0.002 ^b^	4.67 ± 0.00 ^b^	2.41 ± 0.00 ^b^	81.03 ± 0.60 ^g^
X_12_	3	26.19 ± 0.41 ^b^	20.41 ± 0.20 ^c^	35.65 ± 0.10 ^g^	3.77 ± 0.02 ^e^	0.687 ± 0.002 ^c^	4.43 ± 0.00 ^c^	2.29 ± 0.00 ^c^	82.95 ± 0.77 ^f^
X_13_	4	25.67 ± 0.68 ^b^	21.33 ± 0.22 ^bc^	36.91 ± 0.53 ^f^	4.31 ± 0.06 ^d^	0.616 ± 0.003 ^d^	4.26 ± 0.00 ^d^	2.20 ± 0.00 ^d^	85.64 ± 0.75 ^e^
X_14_	5	24.79 ± 0.66 ^c^	20.82 ± 0.49 ^bc^	40.39 ± 0.67 ^d^	4.78 ± 0.06 ^c^	0.556 ± 0.003 ^e^	4.05 ± 0.00 ^e^	2.15 ± 0.00 ^e^	86.95 ± 0.56 ^d^
X_15_	6	20.49 ± 0.10 ^d^	21.04 ± 0.18 ^bc^	39.65 ± 0.33 ^e^	4.74 ± 0.03 ^c^	0.525 ± 0.002 ^f^	3.40 ± 0.00 ^i^	1.88 ± 0.00 ^i^	87.81 ± 1.08 ^c^
X_16_	7	19.75 ± 0.43 ^e^	23.03 ± 0.44 ^a^	43.22 ± 0.44 ^b^	4.92 ± 0.02 ^b^	0.506 ± 0.002 ^g^	3.56 ± 0.00 ^h^	1.91 ± 0.00 ^h^	91.28 ± 0.94 ^a^
X_17_	8	14.35 ± 0.34 ^f^	21.69 ± 0.66 ^b^	42.27 ± 0.20 ^c^	5.03 ± 0.04 ^a^	0.505 ± 0.002 ^g^	3.71 ± 0.00 ^f^	1.93 ± 0.00 ^f^	90.33 ± 0.55 ^b^
X_18_	9	11.37 ± 0.25 ^g^	22.61 ± 0.70 ^a^	44.02 ± 0.11 ^a^	5.04 ± 0.04 ^a^	0.504 ± 0.002 ^g^	3.67 ± 0.00 ^g^	1.94 ± 0.00 ^g^	88.15 ± 0.66 ^c^

Values (mean ± SD) in each column followed by a different letter are significantly different (*p* < 0.05); NPS: normal-pressure steaming.

**Table 3:** **Effect** of processing cycles on the quality indicators of black sesame seeds treated with SHPS.

Sample	Cycle	Water (g/100 g)	Protein (g/100 g)	Fat (g/100 g)	Ash (g/100 g)	Melanin (Abs)	Sesamin (mg/g)	Sesamolin (mg/g)	Sensory Score (Point)
X_19_	1	33.50 ± 0.41 ^a^	21.34 ± 0.41 ^b^	29.22 ± 0.59 ^f^	3.74 ± 0.06 ^c^	0.796 ± 0.002 ^a^	5.75 ± 0.00 ^a^	2.39 ± 0.00 ^a^	80.76 ± 1.07 ^e^
X_20_	2	30.20 ± 0.94 ^b^	21.66 ± 0.28 ^b^	31.73 ± 0.47 ^e^	4.27 ± 0.05 ^b^	0.777 ± 0.002 ^b^	5.12 ± 0.00 ^f^	2.24 ± 0.00 ^b^	81.28 ± 0.57 ^e^
X_21_	3	24.14 ± 0.45 ^c^	23.01 ± 0.20 ^a^	36.07± 0.49 ^d^	4.95 ± 0.06 ^a^	0.774 ± 0.002 ^c^	5.01 ± 0.00 ^h^	2.12 ± 0.00 ^d^	83.11 ± 0.92 ^d^
X_22_	4	22.58 ± 0.04 ^d^	23.44 ± 0.14 ^a^	35.35 ± 0.49 ^d^	4.85 ± 0.04 ^a^	0.773 ± 0.003 ^c^	5.10 ± 0.00 ^g^	2.09 ± 0.00 ^e^	84.02 ± 0.67 ^c^
X_23_	5	17.34 ± 0.07 ^e^	23.43 ± 0.14 ^a^	37.07 ± 0.48 ^c^	4.93 ± 0.01 ^a^	0.773 ± 0.001 ^c^	5.31 ± 0.00 ^b^	2.22 ± 0.00 ^c^	86.33 ± 0.53 ^b^
X_24_	6	15.87 ± 0.71 ^f^	22.68 ± 0.16 ^a^	40.29 ± 0.25 ^b^	5.07 ± 0.11 ^a^	0.765 ± 0.002 ^d^	4.84 ± 0.00 ^i^	1.98 ± 0.00 ^f^	86.58 ± 0.77 ^b^
X_25_	7	13.20 ± 0.34 ^g^	23.20 ± 0.45 ^a^	41.03 ± 0.67 ^b^	4.93 ± 0.01 ^a^	0.765 ± 0.002 ^d^	5.21 ± 0.00 ^c^	2.09 ± 0.00 ^e^	88.64 ± 0.71 ^a^
X_26_	8	10.82 ± 0.32 ^h^	22.00 ± 0.68 ^b^	40.65 ± 0.72 ^b^	5.03 ± 0.10 ^a^	0.764 ± 0.002 ^d^	5.14 ± 0.00 ^d^	1.95 ± 0.00 ^g^	87.19 ± 1.02 ^b^
X_27_	9	8.64 ± 0.30 ^i^	22.92 ± 0.48 ^a^	42.14 ± 0.19 ^a^	5.01 ± 0.18 ^a^	0.764 ± 0.002 ^d^	5.13 ± 0.00 ^e^	1.94 ± 0.00 ^h^	86.62 ± 0.65 ^b^

Values (mean ± SD) in each column followed by a different letter are significantly different (*p* < 0.05); SHPS: high-pressure steaming after soaking in water.

**Table 4:** Effect of processing **cycles** on the quality indicators of black sesame seeds treated with HPS.

Sample	Cycle	Water (g/100 g)	Protein (g/100 g)	Fat (g/100 g)	Ash (g/100 g)	Melanin (Abs)	Sesamin (mg/g)	Sesamolin (mg/g)	Sensory Score (Point)
X_28_	1	27.64 ± 0.52 ^a^	23.74 ± 0.17 ^b^	31.09 ± 0.10 ^e^	4.94 ± 0.00 ^a^	0.814 ± 0.002 ^a^	5.66 ± 0.00 ^a^	2.76 ± 0.00 ^a^	81.38 ± 0.75 ^h^
X_29_	2	24.29 ± 0.68 ^b^	23.66 ± 0.21 ^b^	30.86 ± 0.38 ^e^	4.99 ± 0.04 ^a^	0.806 ± 0.002 ^b^	4.70 ± 0.00 ^f^	2.36 ± 0.00 ^c^	82.19 ± 1.14 ^g^
X_30_	3	23.72 ± 0.22 ^b^	23.62 ± 0.36 ^b^	40.82 ± 0.64 ^d^	5.03 ± 0.04 ^a^	0.800 ± 0.002 ^c^	5.04 ± 0.00 ^b^	2.40 ± 0.00 ^b^	84.03 ± 1.09 ^f^
X_31_	4	22.30 ± 0.24 ^c^	24.77 ± 0.13 ^a^	40.32 ± 0.48 ^d^	5.02 ± 0.04 ^a^	0.793 ± 0.002 ^d^	4.20 ± 0.00 ^i^	2.04 ± 0.00 ^f^	84.98 ± 0.79 ^e^
X_32_	5	19.90 ± 0.01 ^d^	24.77 ± 0.11 ^a^	47.27 ± 0.27 ^c^	4.99 ± 0.07 ^a^	0.791 ± 0.002 ^e^	4.50 ± 0.00 ^h^	2.10 ± 0.00 ^e^	87.96 ± 0.70 ^d^
X_33_	6	14.64 ± 0.12 ^e^	23.53 ± 0.13 ^b^	52.13 ± 0.33 ^b^	5.07 ± 0.03 ^a^	0.784 ± 0.002 ^f^	4.89 ± 0.00 ^d^	2.14 ± 0.00 ^d^	89.97 ± 1.09 ^bc^
X_34_	7	13.92 ± 0.86 ^e^	24.80 ± 0.11 ^a^	53.04 ± 0.45 ^a^	5.05 ± 0.03 ^a^	0.774 ± 0.001 ^g^	4.92 ± 0.00 ^c^	2.04 ± 0.00 ^f^	92.44 ± 0.73 ^a^
X_35_	8	5.53 ± 0.08 ^f^	22.33 ± 0.22 ^c^	51.56 ± 0.39 ^b^	5.09 ± 0.02 ^a^	0.774 ± 0.002 ^g^	4.79 ± 0.00 ^e^	1.99 ± 0.00 ^g^	90.37 ± 0.66 ^b^
X_36_	9	4.92 ± 0.28 ^f^	22.37 ± 0.43 ^c^	51.94 ± 0.11 ^b^	5.10 ± 0.14 ^a^	0.773 ± 0.002 ^g^	4.69 ± 0.00 ^g^	1.88 ± 0.00 ^h^	89.35 ± 0.90 ^c^

Values (mean ± SD) in each column followed by a different letter are significantly different (*p* < 0.05); HPS: high-pressure steaming.

**Table 5:** Effect of **processing** methods on the quality indicators of black sesame seeds treated for nine cycles.

Indicators	Water (g/100 g)	Protein (g/100 g)	Fat (g/100 g)	Ash (g/100 g)	Melanin (Abs)	Sesamin (mg/g)	Sesamolin (mg/g)	Sensory Score (Point)
SNPS	5.09 ± 0.44 ^c^	21.25 ± 0.20 ^b^	42.10 ± 0.26 ^c^	5.24 ± 0.05 ^a^	0.505 ± 0.002 ^c^	3.58 ± 0.00 ^d^	1.91 ± 0.00 ^c^	85.14 ± 0.90 ^d^
NPS	11.37 ± 0.25 ^a^	22.61 ± 0.70 ^a^	44.02 ± 0.11 ^b^	5.04 ± 0.04 ^a^	0.504 ± 0.002 ^c^	3.67 ± 0.00 ^c^	1.94 ± 0.00 ^b^	88.15 ± 0.66 ^b^
SHPS	8.64 ± 0.30 ^b^	22.92 ± 0.48 ^a^	42.14 ± 0.19 ^c^	5.01 ± 0.18 ^a^	0.764 ± 0.002 ^b^	5.13 ± 0.00 ^a^	1.94 ± 0.00 ^a^	86.62 ± 0.65 ^c^
HPS	4.92 ± 0.28 ^c^	22.37 ± 0.43 ^a^	51.94 ± 0.11 ^a^	5.10 ± 0.14 ^a^	0.773 ± 0.002 ^a^	4.69 ± 0.00 ^b^	1.88 ± 0.00 ^d^	89.35 ± 0.90 ^a^

Values (mean ± SD) in each column followed by a different letter are significantly different (*p* < 0.05).

**Table 6:** Weights of various indicators used in the comprehensive evaluation of black sesame seeds.

Indicators	Water	Protein	Fat	Ash	Melanin	Sesamin	Sesamolin	Sensory Score
Mean value	21.304	22.29	39.940	4.561	0.690	4.528	2.128	85.339
Standard deviation	9.014	1.454	6.131	0.634	0.118	0.626	0.205	3.632
*Vi*	0.423	0.065	0.154	0.139	0.170	0.138	0.096	0.043
*Wi*	0.344	0.053	0.125	0.113	0.139	0.113	0.078	0.035

**Table 7:** Standardized data of evaluation indices.

Sample	Treatment	Cycle	Water	Protein	Fat	Ash	Melanin	Sesamin	Sesamolin	Sensory Score
X_0_	Ideal process		1.000	1.000	1.000	1.000	1.000	1.000	1.000	1.000
X_1_	SNPS	1	8.040	0.884	0.640	0.560	0.952	0.781	0.830	0.848
X_2_		2	7.365	0.853	0.644	0.665	0.865	0.739	0.782	0.857
X_3_		3	7.189	0.819	0.658	0.714	0.854	0.774	0.813	0.872
X_4_		4	6.099	0.795	0.689	0.677	0.707	0.736	0.774	0.905
X_5_		5	6.018	0.799	0.792	0.706	0.668	0.745	0.788	0.910
X_6_		6	4.722	0.792	0.823	0.752	0.656	0.645	0.691	0.923
X_7_		7	4.587	0.981	0.844	0.863	0.633	0.607	0.671	0.942
X_8_		8	2.275	0.898	0.820	0.972	0.620	0.647	0.706	0.941
X_9_		9	1.034	0.857	0.794	1.000	0.620	0.623	0.691	0.921
X_10_	NPS	1	6.667	0.922	0.637	0.665	0.965	0.875	0.917	0.869
X_11_		2	5.333	0.849	0.630	0.732	0.908	0.813	0.872	0.877
X_12_		3	5.324	0.823	0.672	0.720	0.843	0.770	0.830	0.897
X_13_		4	5.218	0.860	0.696	0.823	0.756	0.741	0.795	0.926
X_14_		5	5.040	0.839	0.762	0.912	0.682	0.704	0.779	0.941
X_15_		6	4.165	0.848	0.748	0.904	0.645	0.591	0.681	0.950
X_16_		7	4.015	0.929	0.815	0.940	0.621	0.619	0.690	0.987
X_17_		8	2.917	0.875	0.797	0.960	0.620	0.646	0.701	0.977
X_18_		9	2.311	0.912	0.830	0.962	0.619	0.639	0.702	0.954
X_19_	SHPS	1	6.811	0.861	0.551	0.713	0.977	1.000	0.866	0.874
X_20_		2	6.139	0.873	0.598	0.815	0.954	0.890	0.811	0.879
X_21_		3	4.908	0.928	0.680	0.945	0.950	0.871	0.767	0.899
X_22_		4	4.591	0.945	0.666	0.925	0.949	0.887	0.757	0.909
X_23_		5	3.524	0.945	0.699	0.941	0.949	0.924	0.806	0.934
X_24_		6	3.226	0.915	0.760	0.967	0.939	0.842	0.715	0.937
X_25_		7	2.684	0.935	0.774	0.941	0.939	0.906	0.757	0.959
X_26_		8	2.200	0.887	0.766	0.960	0.938	0.893	0.708	0.943
X_27_		9	1.756	0.924	0.795	0.956	0.938	0.893	0.703	0.937
X_28_	HPS	1	5.619	0.957	0.586	0.942	1.000	0.985	1.000	0.880
X_29_		2	4.939	0.954	0.582	0.952	0.989	0.817	0.855	0.889
X_30_		3	4.823	0.952	0.770	0.960	0.983	0.877	0.869	0.909
X_31_		4	4.534	0.999	0.760	0.958	0.974	0.730	0.739	0.919
X_32_		5	4.046	0.999	0.891	0.951	0.971	0.783	0.762	0.952
X_33_		6	2.976	0.949	0.983	0.968	0.962	0.851	0.775	0.973
X_34_		7	2.830	1.000	1.000	0.964	0.950	0.855	0.739	1.000
X_35_		8	1.125	0.900	0.972	0.972	0.950	0.834	0.719	0.978
X_36_		9	1.000	0.902	0.979	0.973	0.949	0.816	0.680	0.967

**Table 8:** Absolute value between reference sequence and comparative sequence.

Sample	Treatment	Cycle	Water	Protein	Fat	Ash	Melanin	Sesamin	Sesamolin	Sensory Score
X_1_	SNPS	1	7.040	0.116	0.360	0.440	0.048	0.219	0.170	0.152
X_2_		2	6.365	0.147	0.356	0.335	0.135	0.261	0.218	0.143
X_3_		3	6.189	0.181	0.342	0.286	0.146	0.226	0.187	0.128
X_4_		4	5.099	0.205	0.311	0.323	0.293	0.264	0.226	0.095
X_5_		5	5.018	0.201	0.208	0.294	0.332	0.255	0.212	0.090
X_6_		6	3.722	0.208	0.177	0.248	0.344	0.355	0.309	0.077
X_7_		7	3.587	0.019	0.156	0.137	0.367	0.393	0.329	0.058
X_8_		8	1.275	0.102	0.180	0.028	0.380	0.353	0.294	0.059
X_9_		9	0.034	0.143	0.206	0.000	0.380	0.377	0.309	0.079
X_10_	NPS	1	5.667	0.078	0.363	0.335	0.035	0.125	0.083	0.131
X_11_		2	4.333	0.151	0.370	0.268	0.092	0.187	0.128	0.123
X_12_		3	4.324	0.177	0.328	0.280	0.157	0.230	0.170	0.103
X_13_		4	4.218	0.140	0.304	0.177	0.244	0.259	0.205	0.074
X_14_		5	4.040	0.161	0.238	0.088	0.318	0.296	0.221	0.059
X_15_		6	3.165	0.152	0.252	0.096	0.355	0.409	0.319	0.050
X_16_		7	3.015	0.071	0.185	0.060	0.379	0.381	0.310	0.013
X_17_		8	1.917	0.125	0.203	0.040	0.380	0.354	0.299	0.023
X_18_		9	1.311	0.088	0.170	0.038	0.381	0.361	0.298	0.046
X_19_	SHPS	1	5.811	0.139	0.449	0.287	0.023	0.000	0.134	0.126
X_20_		2	5.139	0.127	0.402	0.185	0.046	0.110	0.189	0.121
X_21_		3	3.908	0.072	0.320	0.055	0.050	0.129	0.233	0.101
X_22_		4	3.591	0.055	0.334	0.075	0.051	0.113	0.243	0.091
X_23_		5	2.524	0.055	0.301	0.059	0.051	0.076	0.194	0.066
X_24_		6	2.226	0.085	0.240	0.033	0.061	0.158	0.285	0.063
X_25_		7	1.684	0.065	0.226	0.059	0.061	0.094	0.243	0.041
X_26_		8	1.200	0.113	0.234	0.040	0.062	0.107	0.292	0.057
X_27_		9	0.756	0.076	0.205	0.044	0.062	0.107	0.297	0.063
X_28_	HPS	1	4.619	0.043	0.414	0.058	0.000	0.015	0.000	0.120
X_29_		2	3.939	0.046	0.418	0.048	0.011	0.183	0.145	0.111
X_30_		3	3.823	0.048	0.230	0.040	0.017	0.123	0.131	0.091
X_31_		4	3.534	0.001	0.240	0.042	0.026	0.270	0.261	0.081
X_32_		5	3.046	0.001	0.109	0.049	0.029	0.217	0.238	0.048
X_33_		6	1.976	0.051	0.017	0.032	0.038	0.149	0.225	0.027
X_34_		7	1.830	0.000	0.000	0.036	0.050	0.145	0.261	0.000
X_35_		8	0.125	0.100	0.028	0.028	0.050	0.166	0.281	0.022
X_36_		9	0.000	0.098	0.021	0.027	0.051	0.184	0.320	0.033

**Table 9:** Grey-relational coefficient and grey-correlation degree between the reference sequence and comparative sequence.

Sample	Treatment	Cycle	Water	Protein	Fat	Ash	Melanin	Sesamin	Sesamolin	Sensory Score	GCD	GW	GB
X_1_	SNPS	1	0.333	0.968	0.907	0.889	0.986	0.942	0.954	0.959	0.731	9	36
X_2_		2	0.356	0.960	0.908	0.913	0.963	0.931	0.942	0.961	0.736	8	35
X_3_		3	0.363	0.951	0.912	0.925	0.960	0.940	0.950	0.965	0.741	7	34
X_4_		4	0.408	0.945	0.919	0.916	0.923	0.930	0.940	0.974	0.750	6	33
X_5_		5	0.412	0.946	0.944	0.923	0.914	0.932	0.943	0.975	0.754	5	32
X_6_		6	0.486	0.944	0.952	0.934	0.911	0.908	0.919	0.979	0.777	4	25
X_7_		7	0.495	0.995	0.958	0.963	0.906	0.900	0.915	0.984	0.785	3	23
X_8_		8	0.734	0.972	0.951	0.992	0.903	0.909	0.923	0.983	0.870	2	6
X_9_		9	0.990	0.961	0.945	1.000	0.903	0.903	0.919	0.978	0.956	1	3
X_10_	NPS	1	0.383	0.978	0.907	0.913	0.990	0.966	0.977	0.964	0.757	9	30
X_11_		2	0.448	0.959	0.905	0.929	0.974	0.949	0.965	0.966	0.775	6	26
X_12_		3	0.449	0.952	0.915	0.926	0.957	0.939	0.954	0.972	0.771	8	28
X_13_		4	0.455	0.962	0.920	0.952	0.935	0.931	0.945	0.980	0.773	7	27
X_14_		5	0.466	0.956	0.937	0.976	0.917	0.922	0.941	0.983	0.778	5	24
X_15_		6	0.527	0.959	0.933	0.974	0.908	0.896	0.917	0.986	0.792	4	21
X_16_		7	0.539	0.980	0.950	0.983	0.903	0.902	0.919	0.996	0.801	3	17
X_17_		8	0.647	0.966	0.945	0.989	0.903	0.909	0.922	0.994	0.838	2	12
X_18_		9	0.729	0.975	0.954	0.989	0.902	0.907	0.922	0.987	0.868	1	8
X_19_	SHPS	1	0.377	0.962	0.887	0.925	0.994	1.000	0.963	0.965	0.756	9	31
X_20_		2	0.406	0.965	0.898	0.950	0.987	0.970	0.949	0.967	0.765	8	29
X_21_		3	0.474	0.980	0.917	0.985	0.986	0.965	0.938	0.972	0.794	7	19
X_22_		4	0.495	0.985	0.913	0.979	0.986	0.969	0.935	0.975	0.801	6	18
X_23_		5	0.582	0.984	0.921	0.983	0.986	0.979	0.948	0.982	0.835	5	13
X_24_		6	0.613	0.976	0.936	0.991	0.983	0.957	0.925	0.982	0.843	4	11
X_25_		7	0.676	0.982	0.940	0.983	0.983	0.974	0.935	0.988	0.867	3	9
X_26_		8	0.746	0.969	0.938	0.989	0.983	0.971	0.923	0.984	0.889	2	5
X_27_		9	0.823	0.979	0.945	0.988	0.983	0.970	0.922	0.982	0.917	1	4
X_28_	HPS	1	0.432	0.988	0.895	0.984	1.000	0.996	1.000	0.967	0.787	9	22
X_29_		2	0.472	0.987	0.894	0.987	0.997	0.951	0.960	0.969	0.793	8	20
X_30_		3	0.479	0.987	0.939	0.989	0.995	0.966	0.964	0.975	0.803	7	16
X_31_		4	0.499	1.000	0.936	0.988	0.993	0.929	0.931	0.978	0.803	6	15
X_32_		5	0.536	1.000	0.970	0.986	0.992	0.942	0.937	0.986	0.822	5	14
X_33_		6	0.640	0.986	0.995	0.991	0.989	0.959	0.940	0.992	0.863	4	10
X_34_		7	0.658	1.000	1.000	0.990	0.986	0.960	0.931	1.000	0.869	3	7
X_35_		8	0.966	0.973	0.992	0.992	0.986	0.955	0.926	0.994	0.972	2	2
X_36_		9	1.000	0.973	0.994	0.992	0.986	0.950	0.917	0.991	0.983	1	1

GCD: grey-correlation degree; GW: correlation grade within a group; GB: correlation grade between groups.

## Data Availability

The data presented in this study are available on request from the corresponding author.

## Supplementary Materials

The following supporting information can be downloaded at: https://www.mdpi.com/article/10.3390/molecules28135266/s1. Supplementary File S1: Figure S1. Curve of sesamin standard. Figure S2. Curve of sesamolin standard. Supplementary File S2: Table S1. Sensory scoring criteria.

## Sample Availability

Samples of the compounds are not available from the authors.
