# Supplementary material for: Evaluation of Steaming and Drying of Black Sesame Seeds for Nine Cycles Using Grey-Correlation Analysis Based on Variation-Coefficient Weight
Source: Molecules. 2023 Jul 7;28(13):5266. doi: 10.3390/molecules28135266 (PMC10343377; doi:10.3390/molecules28135266)
Supplement: Supplementary file 1 [file molecules-28-05266-s001.zip › Supplementary File S1 (Figures S1 and S2).pptx]

## Slide 1
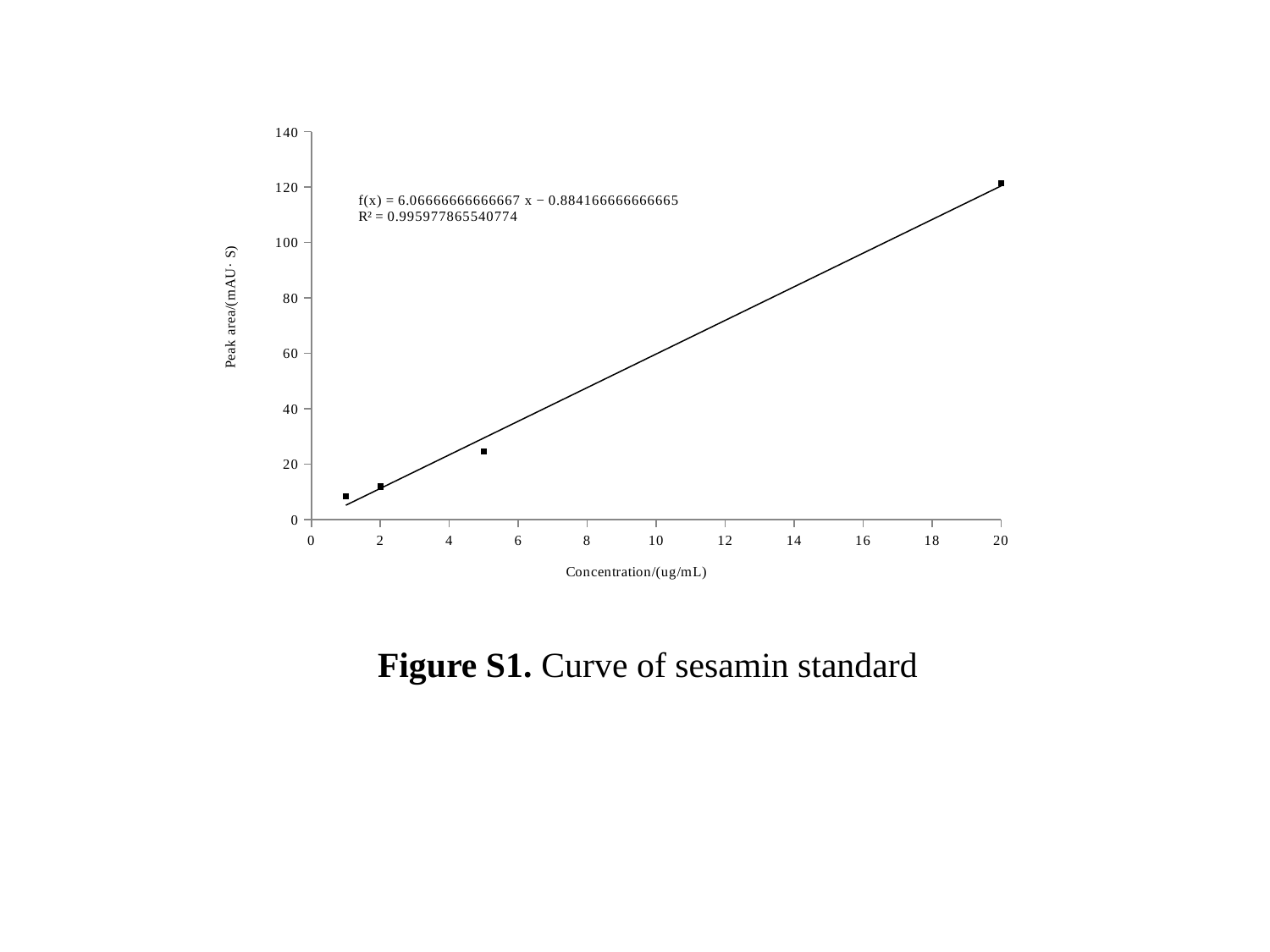

### Chart
| Category | |
|---|---|Figure S1. Curve of sesamin standard

## Slide 2
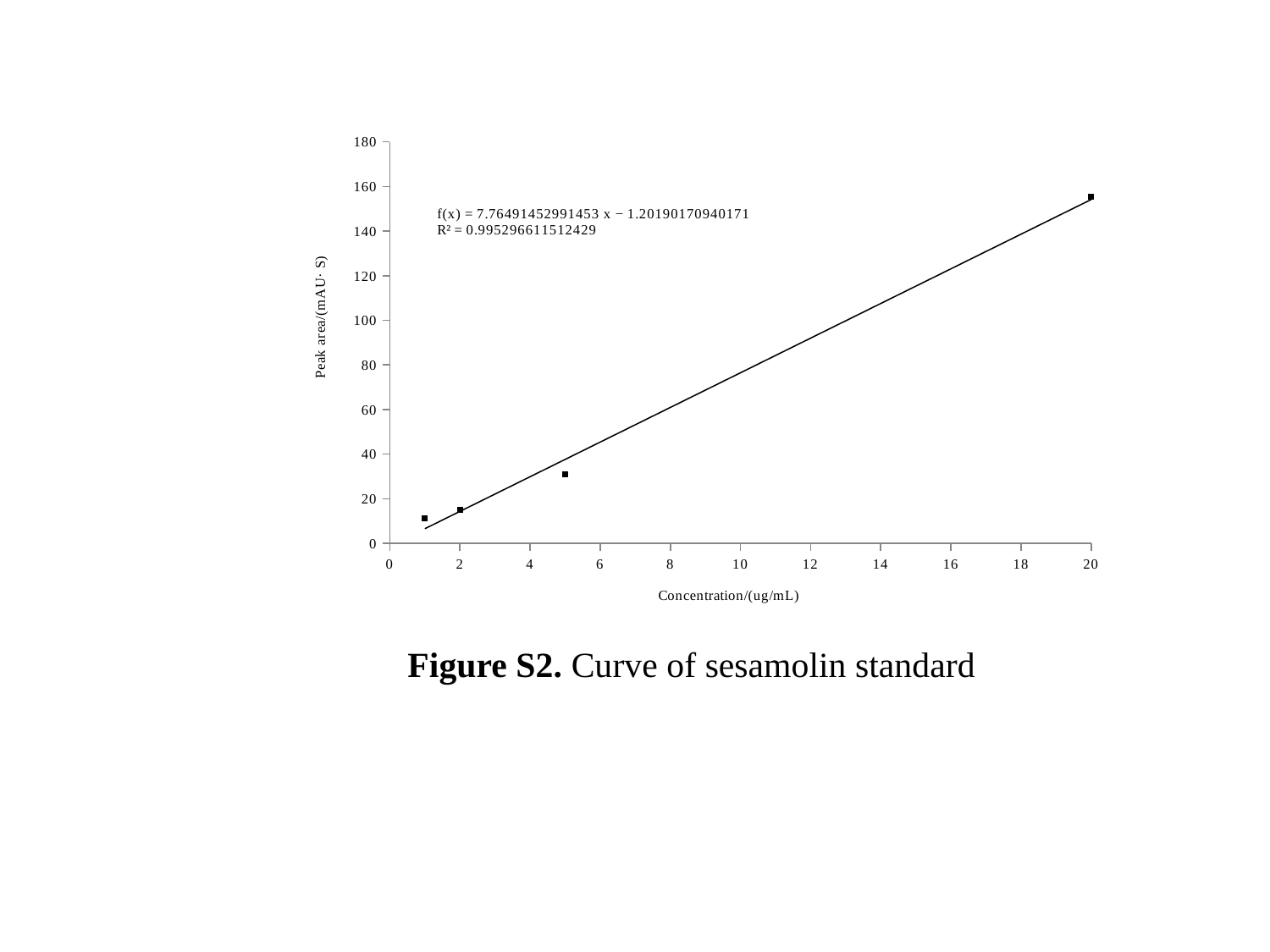

### Chart
| Category | |
|---|---|Figure S2. Curve of sesamolin standard
