# Supplementary material for: Evaluation of Steaming and Drying of Black Sesame Seeds for Nine Cycles Using Grey-Correlation Analysis Based on Variation-Coefficient Weight
Source: Molecules. 2023 Jul 7;28(13):5266. doi: 10.3390/molecules28135266 (PMC10343377; doi:10.3390/molecules28135266)
Supplement: Supplementary file 1 [file molecules-28-05266-s001.zip › Supplementary File S2 (Table S1).pdf]

Table S1. Sensory scoring criteria

Factors	Grading criteria	Sensory score/point
Morphology (100 points)	Smooth surface, no obvious cracks, and no visible impurities in normal vision;	81-100
	The surface is not very round, slightly rough, with inconspicuous graininess and cracks; and no visible impurities in normal vision;	61-80
	Rough surface, with obvious graininess and cracks and visible impurities with normal vision.	Under 60
Color (100 points)	Uniform color, black, and shiny;	81-100
	Color is more even, black, and lustre feeling is weaker;	61-80
	Uneven color, black is not pure, and no luster.	Under 60
Texture (100 points)	Round solid, tight and not loose, and suitable bonding;	81-100
	Round solid, compact, and low adhesion;	61-80
	Poor molding, loose, and coarse powder.	Under 60
Flavor (100 points)	Sesame flavor, pure, and no smell;	81-100
	Light sesame flavor, no smell, and taste sour or sweet;	61-80
	No sesame flavor, odor, and too sour or too sweet.	Under 60
